# Accuracy of blood-glucose measurements using glucose meters and arterial blood gas analyzers in critically ill adult patients: systematic review

**DOI:** 10.1186/cc12567

**Published:** 2013-03-18

**Authors:** Shigeaki Inoue, Moritoki Egi, Joji Kotani, Kiyoshi Morita

**Affiliations:** 1 Department of Emergency and Critical Care Medicine, Tokai University, School of Medicine, 143 Shimokasuya, Isehara-shi, Kanagawa, 259-1193, Japan; 2 Department of Anesthesiology and Resuscitology, Okayama University Hospital, 2-5-1 Shikatachou, Okayama, Okayama 700-8525, Japan; 3 Department of Emergency and Critical Care Medicine, Hyogo College of Medicine, 1-1, Mukogawa-cho, Nishinomiya, Hyogo, 663-8501 Japan

## Abstract

**Introduction:**

Glucose control to prevent both hyperglycemia and hypoglycemia is important in an intensive care unit. Arterial blood gas analyzers and glucose meters are commonly used to measure blood-glucose concentration in an intensive care unit; however, their accuracies are still unclear.

**Methods:**

We performed a systematic literature search (January 1, 2001, to August 31, 2012) to find clinical studies comparing blood-glucose values measured with glucose meters and/or arterial blood gas analyzers with those simultaneously measured with a central laboratory machine in critically ill adult patients.

**Results:**

We reviewed 879 articles and found 21 studies in which the accuracy of blood-glucose monitoring by arterial blood gas analyzers and/or glucometers by using central laboratory methods as references was assessed in critically ill adult patients. Of those 21 studies, 11 studies in which International Organization for Standardization criteria, error-grid method, or percentage of values within 20% of the error of a reference were used were selected for evaluation. The accuracy of blood-glucose measurements by arterial blood gas analyzers and glucose meters by using arterial blood was significantly higher than that of measurements with glucose meters by using capillary blood (odds ratios for error: 0.04, *P *< 0.001; and 0.36, *P *< 0.001). The accuracy of blood-glucose measurements with arterial blood gas analyzers tended to be higher than that of measurements with glucose meters by using arterial blood (*P *= 0.20). In the hypoglycemic range (defined as < 81 mg/dl), the incidence of errors using these devices was higher than that in the nonhypoglycemic range (odds ratios for error: arterial blood gas analyzers, 1.86, *P *= 0.15; glucose meters with capillary blood, 1.84, *P *= 0.03; glucose meters with arterial blood, 2.33, *P *= 0.02). Unstable hemodynamics (edema and use of a vasopressor) and use of insulin were associated with increased error of blood glucose monitoring with glucose meters.

**Conclusions:**

Our literature review showed that the accuracy of blood-glucose measurements with arterial blood gas analyzers was significantly higher than that of measurements with glucose meters by using capillary blood and tended to be higher than that of measurements with glucose meters by using arterial blood. These results should be interpreted with caution because of the large variation of accuracy among devices. Because blood-glucose monitoring was less accurate within or near the hypoglycemic range, especially in patients with unstable hemodynamics or receiving insulin infusion, we should be aware that current blood glucose-monitoring technology has not reached a high enough degree of accuracy and reliability to lead to appropriate glucose control in critically ill patients.

## Introduction

Glucose control to prevent both hyperglycemia and hypoglycemia is important in an intensive care unit [1]. Recent meta-analysis, including results of the NICE-SUGAR study [2], showed that intensive insulin therapy (target blood-glucose control, 80 to 110 mg/dl) was not beneficial and increased the risk of severe hypoglycemia in critically ill patients [3-5]. Thus, it is currently recommended that insulin should be used when the glucose concentration exceeds 180 mg/dl, and target glucose concentration should generally be between 144 and 180 mg/dl [6,7]

Even though a more-modest target for blood-glucose concentration is now accepted, the importance of glucose monitoring and its accuracy has become clearer. Because the physiological activity of glucose is dependent on its plasma concentration, central laboratory blood-glucose measurement using plasma (Glu-lab) is recommended [8,9]. However, arterial blood gas analyzers (ABGs) and/or glucose meters, not Glu-lab, are commonly used to measure blood-glucose concentrations in critically ill patients, because of their convenience and speed [10]. Because most of these devices were not developed to guide the administration of insulin in critically ill patients, they might not be sufficiently accurate to guide therapy aimed at maintaining blood glucose within a 30-mg/dl range [11]. Therefore, knowledge of their limitations is essential to minimize the possibility of a harmful measurement error. However, no systematic literature review has assessed the agreement of measurements by ABGs and/or glucose meters in critically ill patients.

Accordingly, we performed a systematic review and meta-analysis of selected observational studies on the accuracy of blood-glucose measurements by using ABGs (Glu-ABGs), glucose meters using capillary blood samples (Gluco-C), and glucose meters using arterial blood samples (Gluco-A) in critically ill adult patients.

## Materials and methods

### Electronic database

We performed a systematic literature search (January 1, 2001, through August 31, 2012) to find clinical studies comparing blood-glucose values measured by using ABGs and/or glucose meters with those simultaneously measured with a central laboratory machine in critically ill adult patients. The literature search was performed by using MEDLINE and PubMed electronic databases with the following key words: "intensive care", "critical care," "glucose," "sugar," "glycemic," "insulin," "Bland Altman," "agreement," "validation," "reliability," "accuracy," "correlation," "Clarke grid," and "bias." All articles identified by this search strategy were obtained, and their bibliographies were studied for articles that might have been missed by the electronic database search.

### Inclusion and exclusion criteria

Inclusion criteria for the current systematic review were as follows: (a) studies conducted in critically ill adult patients, (b) studies in which the accuracy of glucose monitoring was assessed by using ABGs and/or glucose meters, (c) studies in which Glu-lab values were used as reference values, and (d) articles presenting an appropriate summary of statistics. We excluded nonhuman studies, non-English-language articles, and pediatric studies.

### Data extraction and interpretation

Two of the authors (SI and ME) extracted data from selected articles, which were then reviewed by coauthors. We paid particular attention to determine whether the accuracy of blood-glucose monitoring was influenced by types of devices and sites of blood collection. Because the accuracy of blood-glucose monitoring in a hypoglycemic range is important, we performed further assessment of accuracy in a hypoglycemic range, defined as < 81 mg/dl. Additionally, we summarized factors associated with errors of blood-glucose measurements.

### Outcomes

#### Primary outcome

Most of the studies were conducted by using (a) agreement (percentages of blood-glucose values with an acceptable error), and/or (b) bias (mean difference between devices and reference) for evaluation.

Because the International Organization for Standardization (ISO) criteria use agreement within ±20% of Glu-lab at or above 75 mg/dl and within ±15 mg/dl below 75 mg/dl, we defined primary outcome as percentages of blood-glucose values within ±20% of the error of Glu-lab, which involved Zone A of error-grid analysis (agreement within ±20% of Glu-lab at or above 70 mg/dl) and agreement with ISO criteria. We obtained rates of overestimation and underestimation of blood-glucose measurements. We defined proportion of nonagreement < 5% as good quality of blood-glucose measurements according to ISO criteria.

#### Secondary outcomes

We obtained the proportion of agreement by using criteria other than the previously described criteria. Because many reports showed the bias of each device, we summarized their bias.

### Statistical analysis

The current systematic review was performed by following the MOOSE statement for observational studies [12]. Analysis was performed by using Review Manager (RevMan) (The Cochrane Collaboration, 2008; The Nordic Cochrane Centre, Copenhagen, Denmark). Heterogeneity was calculated by the I2 test, which shows the rate of variation across studies due to heterogeneity rather than to chance (ranging from 0 (no heterogeneity) to 100 (maximum heterogeneity)) [13]. Given the significant heterogeneity found among the results of the studies, the random-effects model was used [14]. All results are reported with 95% confidence intervals. A *P *value < 0.05 was taken to indicate statistical significance.

## Results

**Figure 1:**
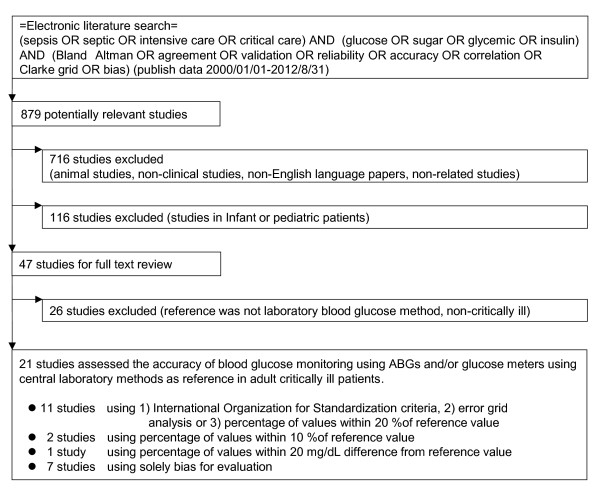
**Study selection for inclusion systematic review for accuracy of glycemic measurements in the critically ill patients**.

We identified 879 potentially relevant articles by the literature search. We excluded 716 studies because they were animal studies, nonclinical studies, non-English-language articles, or nonrelated studies. Of the remaining 163 studies, 116 were excluded because they were performed in infant or pediatric populations. Full text reviews were conducted for the remaining 47 articles. In 21 of those 47 studies, the accuracy of blood-glucose monitoring was assessed by using ABGs and/or glucometers with central laboratory methods as references in critically ill adult patients (Figure 1).

Among the 21 selected studies [15-35], 11 studies [15-25] used ISO criteria, the error-grid method, or percentage of values within 20% of the error of a reference; three studies [26-28] used agreement with criteria other than these criteria, and seven studies [29-35] used only bias for evaluation (Figure 1, Table 1).

**Table 1:** Observational studies to test the accuracy of point of blood-glucose monitoring in critically ill adult patients (January 2001 to August 2012)

First author (year)	Study design	Age (years)	APACHE II	No	Protocol	Central Lab Machine	ABG	Gluco-C	Gluco-A	Agreement (within)	Bias	Ref
Stadlbauer V (2010)	Pro	55	17	17	-	Hexokinase method	○	-	-	20%	Yes	[15]

Corstjens AM (2006)	Pro	(32-88)	-	45	Every 6 hours	YSI2300	○	-	-	20%	Yes	[16]

Hoedemaekers CW (2008)	Pro	-	-	32	1/pts	Aeroset	○	-	-	20%	Yes	[17]

Slater-MacLean L (2008)	Pro	56	19	60	3 per day	YSI2300	○	○	○	20%	Yes	[18]

Kanji S (2005)	Pro	68	22	30	Every 5:00	LX-20	○	○	○	20%	No	[19]

Petersen JR (2008)	Retro	-	-	84	1/pts	Vitros950 or 5.1FS	○	○	○	20%	Yes	[20]

Desachy A (2008)	Pro	59	-	85	First 4 times	Dimension Vista device	-	○	○	20%	Yes	[21]

Pulzi Júnior SA (2009)	Retro	55	16	40	1/pts	Au640e	-	○	○	20%	Yes	[22]

Lonjaret L (2012)	Pro	59	-	75	Every 5:00	Glucose oxidase method	-	○	○	20%	Yes	[23]

Critchell CD (2007)	Pro	59	16	80	Every 12 or 24 h	LX-20	-	○	-	20%	Yes	[24]

Meynaar IA (2009)	Pro	72	18	32	Every 4 h	Architect CI8200	-	-	○	20%	Yes	[25]

Cook A (2009)	Pro	58	-	67	1/pts	Au640	-	○	-	20 mg/dl	Yes	[26]

Karon BS (2007)	Pro	69	-	20	First 5 hourly	Double P Modular system	-	○	○	10%	Yes	[27]

Karon (2008)	Pro	-	-	-	-	Roche Integra 400	-	-	○	10%	Yes	[28]

Fekih Hassen M (2010)	Pro	60	-	43	6/pts	Dade-Behring Multichannel Analyzer	-	○	-	-	Yes	[29]

Finkielman JD (2005)	Retro	56	-	197	-	Glucose Analyzer 2 or Hitachi 747-200	-	○	○	-	Yes	[30]

Lacala T (2007)	Pro	67	-	42	1/pts	RxL	-	○	○	-	Yes	[31]

Mann EA (2008)	Pro	-	-	-	-	Vitros Fusion	-	-	○	-	Yes	[32]

Shearer A (2009)	Pro	64	-	63	1/pts	Au640	-	○	-	-	Yes	[33]

Ray JG (2001)	Pro	67	-	10	-	Cobas Integra Analyzer	-	-	○	-	Yes	[34]

Denfeld QE (2011)	Pro	61	-	46	1/pts	DXC 800	-	-	○	-	Yes	[35]

Labo, laboratory; ABG, arterial blood gas analyzer; Gluco-C, glucose meters using capillary blood samples; Gluco-A, glucose meters using arterial blood samples; ○, device evaluated, **-**, not evaluated; pts, patients; Pro, prospective study; Retro, retrospective study.

### Bias of point of blood-glucose monitoring in adult critically ill patients

Bias of point of blood-glucose monitoring in critically ill adult patients was assessed in 20 studies (Table 2). Bias was assessed for Glu-ABGs in five studies [15-18,20], for Gluco-C in 13 studies [18,20-27,29-31,33,34], and for Gluco-A in 12 studies [18,20-23,25,27,28,30-32,35]. The mean differences varied between -2.7 mg/dl [17] and 25.2 mg/dl [16] in Glu-ABGs, between -16 mg/dl [23] and 9.9 mg/dl [22] in Gluco-C, and between -10 mg/dl [23] and 23.0 mg/dl [32] in Gluco-A.

**Table 2:** Bias of point of blood-glucose monitoring in critically ill adult patients

First author (year)	Device (ABG)	Device (Gluco-C)	Device (Gluco-A)	Bias (ABG)	Bias (Gluco-C)	Bias (Gluco-A)	Ref
Stadlbauer V (2010)	Cobas B 221 ABL 800 Grem Premiere 3000	-	-	8.4 (-5.3, 22.1) 7.6 (-1.9, 17.0) 4.6 (-7.9, 17.0)	-	-	[15]

Corstjens AM (2006)	ABL715	-	-	Mean D = 25.2	-	-	[16]

Hoedemaekers CW (2008)	Rapidlab	-	-	-2.7 (-22.3, 16.9)	-	-	[17]

Slater-MacLean L (2008)	Chiron865	SureStepflexx Accu-Chek Inform FreeStyle	SureStepflexx Accu-Chek Inform FreeStyle	Mean D = 0.4	Mean D = 9.2 Mean D = -4.5 Mean D = 5.8	Mean D = 3.4 Mean D = -10.1 Mean D = 1.6	[18]

Kanji S (2005)	RapidLab860	Accu-Chek Inform	Accu-Chek Inform	-	-	-	[19]

Petersen JR (2008)	Rapidpoint405	Accu-Chek Inform	Accu-Chek Inform	Mean D = 1.8	Mean D = 9	Mean D = 12.6	[20]

Desachy A (2008)	-	Accu-Chek Sensor	Accu-Chek Sensor	-	1.5 (-55.3, 58.3)	1.4 (-39.5, 42.4)	[21]

PulziJúnior SA (2009)	-	FreeStyle	FreeStyle	-	9.9 (-52.4, 72.1)	6.8 (-30.6. 44.1)	[22]

Lonjaret L (2012)	-	Contour	Contour	-	-16 (-59.1, 27.1)	-10 (-51.2, 31.2)	[23]

Critchell CD (2007)	-	Accu-Chek Inform	-	-	8.6 (-28.6, 45.8)	-	[24]

Meynaar IA (2009)	-	-	Accu-Chek Inform	-	-	11 (-20.2, 42.2)	[25]

Cook A (2009)	-	SuperStepFlexx	-	-	9.5 (-12.5, 31.5)	-	[26]

Karon BS (2007)	-	Accu-Chek Inform	Accu-Chek Inform	-	Median = -1 [IQR -4, 5]	Median = 14 [IQR 10, 18]	[27]

Karon (2008)	-	-	Accu-Chek Inform Precision PCx SureStepFlexx StatStrip	-	-	Median = -9 Median = -12 Median = 2 Median = -3	[28]

Fekih Hassen M (2010)	-	Accu-Chek	-	-	-0.9 (-74.3, 72.5)	-	[29]

Finkielman JD (2005)	-	SureStepFlexx	SureStepFlexx	-	7.9 (-27.2, 43.1)^a^	7.9 (-27.2, 43.1)^a^	[30]

Lacala T (2007)	-	Sure Step pro	Sure Step Pro	-	1.0 (-23.1, 25.1)	-0.1 (-21.7, 21.5)	[31]

Mann EA (2008)	-	-	Sure Step Flexx Accu-Chek Inform Accu-Chek Advantage Precision PCx	-	-	19.1 (3.7, 34.5) 20.7 (-0.8, 42.2) 22.0 (-0.8, 44.8) 23.0 (1.6, 44.4)	[32]

Shearer A (2009)	-	SureStepFlexx	-	-	8.7 (-18.2, 35.6)	-	[33]

Ray JG (2001)	-	-	One-touch profile	-	-	0.7 (-39.6, 41.4)	[34]

Denfeld QE (2011)	-	-	Precision Xceedpro	-	-	12.3 (-6.9, 31.5)	[35]

Bias was described as mean difference (95% confidence interval). ABG, arterial blood gas analyzer; Gluco-C, glucose meters using capillary blood samples, Gluco-A, glucose meters using arterial blood samples; Ref, reference; Mean D, mean difference; IQR, interquartile range. ^a^Analysis of merged data from glucometer using capillary and arterial samples.

Limits of agreements were shown for Glu-ABGs in two reports [15,17], for Gluco-C in 10 studies [21-24,26,29-31,33,34], and for Gluco-A in seven studies [21-23,25,30-32,35]. Its range (upper limit to lower limit) varied between 19 mg/dl [15] and 39 mg/dl [17] for Glu-ABGs, between 44 mg/dl [26] and 144 mg/dl [29] for Gluco-C, and between 38 mg/dl [32] and 82 mg/dl [23] for Gluco-A.

### Characteristics of 11 inclusion studies

Eleven studies [15-25] that used ISO criteria, error-grid method, or percentage of values within 20% of the error of a reference were selected for further assessment (Table 1). All of the 11 studies were single-center observational studies. Nine of the 11 studies were prospective studies [15-19,21,23-25], and the other two studies were retrospective studies [20,22]. Totally, 580 patients were included in the 11 studies.

Various types of central laboratory machines were used in the studies. The two methods for blood-glucose monitoring are the hexokinase method (Aeroset, Dimention, and Vista device, Au640e and Architect CI 8200) [15,17,21,22,25] and the glucose oxidase method (YSI 2300, Lx-20, Vitros950, and 5.1FS) [16,18-20,23,24]. All machines had traceability to a higher-order reference method.

### Accuracy of blood-glucose measurements in the whole glycemic range

#### Arterial blood glucose analyzers

The accuracy of Glu-ABGs including Cobas B 221 [15], ABL 800 [15], Grem Premiere 3000 [15], ABL715 [16], RapidLab [17], Chiron865 [18], Rapidlab 860 [19], and Rapidpoint 405 [20] was assessed in six studies (Table 3). Arterial blood samples were used in all of those studies. The accuracy of Glu-ABGs was assessed by using ISO criteria in one study [17], error-grid analysis in four studies [15,16,18,20], and 20% error in one study [19].

**Table 3:** Agreement of blood-glucose monitoring with each device

First author (year)	Devices	Methods for assessment	Nonagreement Proportion < 5%	Nonagreement proportion	Overestimation	Underestimation	Ref
ABG							

Stadlbauer V (2010)	Cobas B 221 ABL 800 Grem Premiere 3000	Clarke error grid	Yes	0/74 (0%)	0/74 (0%)	0/74 (0%)	[15]

Corstjens AM (2006)	ABL715	Clarke error grid	No	178/416 (42.8%)	178/416 (42.8%)	0/416 (0%)	[16]

Hoedemaekers CW (2008)	RapidLab	ISO	Yes	0/32 (0%)	0/32 (0%)	0/32 (0%)	[17]

Slater-MacLean L (2008)	Chiron865	Modified error grid	Yes	1/683 (0.1%)	0/683 (0%)	1/683 (0.1%)	[18]

Kanji S (2005)	RapidLab860	Within 20%	Yes	1/115 (0.9%)	0/115 (0%)	1/115 (0.9%)	[19]

Petersen JR (2008)	Rapidpoint405	Modified error grid	Yes	0/114 (0%)	0/114 (0%)	0/114 (0%)	[20]

Total			5/6 (83.3%)	180/1,444 (12.5%)	178/1,444 (12.3%)	2/1,444 (0.1%)	

Gluco-C							

Slater-MacLean L (2008)	SuperStrepFlexx Accu-Chek Inform FreeStyle	Modified error grid	Yes	24/1,656 (1.4%)	15/1,656 (0.9%)	9/1,656 (0.5%)	[18]

Kanji S (2005)	Accu-Chek Inform	Within 20%	No	32/118 (27.1%)	26/118 (22.0%)	6/118 (5.1%)	[19]

Petersen JR (2008)	Accu-Chek Inform	Modified error grid	No	23/114 (20.2%)	20/114 (17.4%)	3/114 (2.6%)	[20]

Desachy A (2008)	Accu-Chek Sensor	Within 20%	No	41/273 (15.0%)	12/273 (4.4%)	29/273 (10.6%)	[21]

PulziJúnior SA (2009)	FreeStyle	Within 20%	No	9/38 (23.4%)	8/38 (21.1%)	1/38 (2.6%)	[22]

Lonjaret L (2012)	Contour	Within 20%	No	75/302 (24.8%)	8/302 (2.6%)	67/302 (22.2%)	[23]

Critchell CD (2007)	Accu-Chek Inform	ISO	No	53/277 (19.1%)	44/277 (15.9%)	9/277 (3.2%)	[24]

Total			1/7 (14.3%)	257/2,778 (9.3%)	133/2,778 (4.8%)	124/2778 (4.5%)	

Gluco-A							

Slater-MacLean L (2008)	SuperStrepFlexx Accu-Chek Inform FreeStyle	Modified error grid	Yes	1/2,048 (0.05%)	0/2,048 (0%)	1/2,048 (0.05%)	[18]

Kanji S (2005)	Accu-Chek Inform	Within 20%	No	14/113 (12.3%)	10/113 (8.8%)	4/113 (3.5%)	[19]

Petersen JR (2008)	Accu-Chek Inform	Modified error grid	No	13/114 (11.3%)	13/114 (11.3%)	0/114 (0%)	[20]

Desachy A (2008)	Accu-Chek Sensor	Within 20%	No	13/232 (5.6%)	n.a.	n.a.	[21]

PulziJúnior SA (2009)	FreeStyle	Within 20%	No	3/38 (7.9%)	3/38 (7.9%)	0/38 (0)	[22]

Lonjaret L (2012)	Contour	Within 20%	No	35/302 (11.6%)	7/302 (2.3%)	28/302 (9.3%)	[23]

Meynaar IA (2009)	Accu-Check Inform	Within 20%	No	22/239 (9.6%)	3/239 (1.3%)	19/239 (7.9%)	[25]

Total			1/7 (14.3%)	101/3,086 (3.3%)	36/2,854 (1.3%)	52/2,854 (1.8%)	

ABG, arterial blood gas analyzer; Gluco-C, glucose meters using capillary blood samples; Gluco-A, glucose meters using arterial blood samples; Ref, reference.

There were 1,444 assessments in the six studies. The proportion of nonagreement varied from 0 to 42.8%. Five (83.3%) studies showed good quality of blood-glucose monitoring (nonagreement, < 5%). The proportion of nonagreement was 12.5% in total. Overestimation of blood-glucose concentrations was seen in 12.3% of all assessments.

#### Glucose meters using capillary blood samples

In seven studies, the accuracy of Gluco-C, including SuperStrepFlexx [18], AccuCheck Inform [18-20,24], FreeStyle [18,22], Accu-Chek Sensor [21], and Contour [23], was assessed (Table 3). The accuracy of Gluco-C was assessed by using ISO criteria in one study [24], error-grid analysis in two studies [18,20], and 20% error in four studies [19,21-23].

In the 2,778 assessments in the seven studies, the proportion of nonagreement varied from 1.4% to 27.1%. One study (14.3%) showed a good quality of blood-glucose monitoring [18]. The proportion of nonagreement was 9.3%. Overestimation of blood-glucose concentrations was seen in 4.8% of all assessments.

#### Glucose meters using arterial blood samples

In seven studies, the accuracy of Gluco-A, including SuperStrepFlexx [18], AccuCheck Inform [18-20,25], FreeStyle [18,22], Accu-Chek Sensor [21], and Contour [23] was assessed (Table 3). The accuracy of Gluco-A was assessed by using error-grid analysis in two studies [18,20] and 20% error in five studies [19,21-23,25].

In the seven studies, 3,086 assessments were done. The proportion of nonagreement varied from 0 to 12.3%. One study (14.3%) showed good quality of blood-glucose monitoring [18]. The proportion of nonagreement was 3.3% (*n *= 101). Overestimation of blood-glucose values was seen in 1.3% of all assessments.

#### Meta-analysis to compare the accuracy of devices

**Figure 2:**
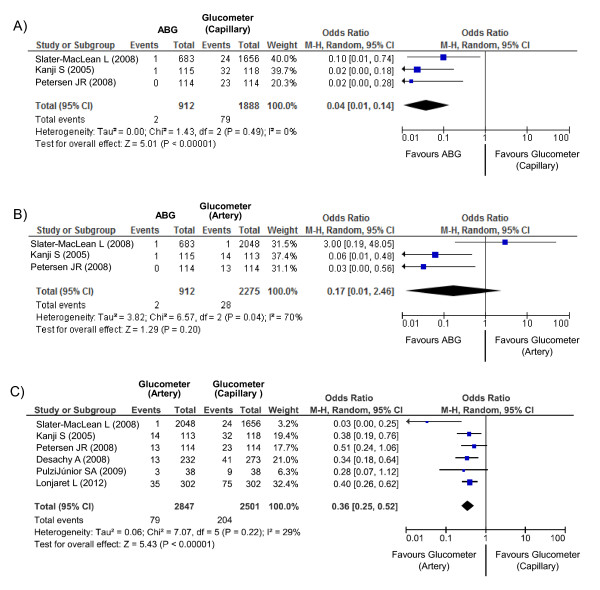
**The comparisons of accuracy of point of blood glucose monitoring**. Size of data markers is proportional to the weight of each study in the forest plot. ABG, arterial blood gas analyzers; CI, confidence interval.

In three studies, the accuracy of ABGs and that of glucose meters were compared simultaneously [18-20]. Glu-ABGs were significantly more accurate than Gluco-C (odds ratio for nonagreement, 0.04; *P *< 0.001) (Figure 2A). Glu-ABGs tended to be more accurate, but not significantly more accurate, than Gluco-A (odds ratio for nonagreement, 0.17; *P *= 0.20) (Figure 2B).

In six studies, the accuracy of Gluco-A and that of Gluco-C [18-23] were compared. Gluco-A results were significantly more accurate than those of Gluco-C (odds ratio for nonagreement, 0.36; *P *< 0.001) (Figure 2C).

### Accuracy of blood-glucose measurements in the hypoglycemic range

The accuracy of point of blood-glucose monitoring in the hypoglycemic range was assessed for Glu-ABGs in two studies [16,19], for Gluco-C in three studies [19,23,24], and for Gluco-A in three studies [19,23,25] (Table 4). The total number of assessments was 157 (59 assessments for ABGs, 52 assessments for Gluco-C, and 46 assessments for Gluco-A).

**Table 4:** Agreement of each method in the hypoglycemic range

First author (year)	Devices	Methods	Definition (mg/dl)	No of samples	Non Agreement proportion < 5%	Non agreement proportion	Over estimation	Under estimation	Non agreement in non-hypo range	Odds ratio (95% CI)	Ref
ABG											

Corstjens AM (2006)	ABL 715	Zone A	70	22	No	13/22 (59%)	13/22 (59%)	0/22 (0)	165/394 (41.9%)	2.00 (0.84, 4.80)	[16]

Kanji S (2005)	RapidLab 860	Within 20%	81	37	Yes	0/37 (0)	0/37 (0)	0/37 (0)	1/78 (1.3%)	0.69 (0.03, 17.3)	[19]

Total				59	1/2 (50%)	13/59 (22.0%)	13/59 (22.0%)	0/59 (0)	166/472 (35.2%)	1.86 (0.80, 4.33)	

Gluco-C											

Kanji S (2005)	Accu-Chek Inform	Within 20%	81	38	No	14/38 (38%)	11/38 (30%)	3/38 (8%)	18/80 (22.5%)	2.30 (1.00, 5.32)	[19]

Lonjaret L (2012)	Contour	Within 20%	81	25	No	8/25 (32%)	3/25 (12%)	5/25 (20%)	67/277 (24.2%)	1.47 (0.61, 3.57)	[23]

Critchell CD (2007)	Accu-Chek Inform	< 15 mg/dl	75	14	No	4/14 (29%)	1/14 (7%)	3/14 (21%)	49/263 (18.6%)	1.75 (0.53, 5.80)	[24]

Total				77	0/3 (0%)	26/77 (33.8%)	15/77 (19.5%)	11/77 (14.3%)	134/620 (21.6%)	1.84 (1.07,3.16)	

Gluco-A											

Kanji S (2005)	Accu-Chek Inform	Within 20%	81	36	No	8/36 (22%)	6/36 (17%)	2/36 (6%)	6/77 (7.8%)	3.38 (1.08, 10.6)	[19]

Lonjaret L (2012)	Contour	Within 20%	81	25	No	5/25 (20%)	1/25 (4%)	4/25 (16%)	30/277 (10.8%)	2.06 (0.72, 5.89)	[23]

Meynaar IA (2009)	Accu-Check Inform	< 15 mg/dl	75	10	No	1/10 (10%)	1/10 (10%)	0/10 (0%)	21/229 (9.2%)	1.10 (0.13, 9.12)	[25]

Total				71	0/3 (0%)	14/71 (19.7%)	8/71 (11.3%)	6/71 (8.4%)	57/583 (9.8%)	2.33 (1.13, 4.83)	

Definition, Definition of hypoglycemia; No of samples, Number of samples in hypoglycemic range; non-hypo, non-hypoglycemic. ABG, arterial blood gas analyzer; Gluco-C, glucose meters using capillary blood samples; Gluco-A, glucose meters using arterial blood samples; CI, confidence interval; Ref, reference.

For ABGs, 13 of the 59 blood-glucose measurements were outside the agreement range (22.0%), and all of them overestimated blood-glucose values (22.0%). One study by Kanji *et al*. [19] showed a high level of accuracy of ABGs in the hypoglycemic range (nonagreement, none of 37) [19]. For Gluco-C, 26 of the 77 blood-glucose measurements were outside the agreement range (33.8%). Overestimation of blood-glucose values was seen in 15 measurements (19.5%). For Gluco-A, 14 of the 71 blood-glucose measurements were outside the agreement range (19.7%). Overestimation of blood-glucose values was seen in eight (11.3%) measurements.

Blood-glucose measurements in the hypoglycemic range were less accurate than were those in the nonhypoglycemic range among all three devices (odds ratio for error, Glu-ABGs, 1.86, *P *= 0.15; Gluco-C, 1.84, *P *= 0.03; Gluco-A, 2.33, *P *= 0.02).

### Factors associated with error of blood-glucose measurements

In six studies, risk factors for inaccuracy of glucose measurements were determined (five for Gluco-C, five for Gluco-A, and none for ABGs) [20-25] (Table 5). Patient's factors (sex, body mass index, severity of illness, and presence of sepsis and/or diabetes), except for age, were not significantly related to inaccuracy. Young age was significantly associated with increased risk of nonagreement for Gluco-C in one study [23]. No laboratory data (albumin, lactate, PaCO_2_, PaO_2_, pH, and hematocrit) were associated with inaccuracy.

**Table 5:** Risk factors for inaccuracy of glucose monitoring

First author (year)	Sex	Age	BMI	Severity of illness	Sepsis	DM	Alb	Lac	PaO_2_	PaCO_2_	pH	Ht	Use of insulin	Use of steroid	**P.I**.	HR	Use of Vaso- pressor	Low peripheral perfusion	Low MAP	Edema	Ref
Gluco-C																					

Petersen JR (2008)	-	-	-	-	-	-	-	-	-	-	-	-	-	-	-	-	-	-	-	+	[20]

Desachy A (2008)	-	-	-	○	-	○	-	-	-	-	-	○	-	-	+	○	-	○	○	-	[21]

PulziJúnior SA (2009)	-	-	-	-	-	-	-	-	-	-	-	-	-	-	-	-	+	○	-	-	[22]

Critchell CD (2007)	○	○	-	○	○	-	○	-	○	○	○	○	-	○	-	-	+	-	-	+	[24]

Lonjaret L (2012)	○	+	○	○	-	-	-	○	-	-	○	-	+	-	-	-	○	-	-	-	[23]

Gluco-A																					

Petersen JR (2008)	-	-	-	-	-	-	-	-	-	-	-	-	-	-	-	-	-	-	-	○	[20]

Desachy A (2008)	-	-	-	○	-	○	-	-	-	-	-	○	-	-	○	○	-	+	+	-	[21]

PulziJúnior SA (2009)	-	-	-	-	-	-	-	-	-	-	-	-	-	-	-	-	○	○	-	-	[22]

Meynaar IA (2009)	-	-	-	-	-	-	-	-	-	-	-	○	-	-	-	-	-	-	-	-	[25]

Lonjaret L (2012)	○	○	○	○	-	-	-	○	-	-	○	-	+	-	-	-	+	-	-	-	[23]

BMI, Body mass index; DM, diabetes mellitus, Alb, serum albumin concentration; Lac, lactate concentration; Ht, hematocrit; P.I, perfusion index, MAP, mean arterial pressure, Ref, reference; Gluco-C, glucose meter; using capillary blood samples; Gluco-A, glucose meters using arterial blood samples. +, factor significantly associated with disagreement of blood glucose monitoring. ○, factor not significantly associated with disagreement of blood glucose monitoring.

For Gluco-C, low perfusion index [36], use of a vasopressor [22,24] and presence of edema [20,24] were significantly associated with inaccuracy. For Gluco-A, use of a vasopressor [23], low peripheral perfusion, and low mean arterial pressure [21] were associated with inaccuracy.

### Studies in which agreement of criteria other than "within 20%" was assessed

Our literature review retrieved three studies in which agreement of criteria other than "within 20%" was assessed: one study used within 20 mg/dl from the reference [26], and two studies used within 10% of reference methods for evaluation [27,28] (Table 6). No study showed a good quality of blood-glucose monitoring. One study (*n *= 20) showed that blood-glucose measurements by Accu-Chek Inform using arterial blood samples were less accurate than those using capillary blood samples (odds ratio for incidence of nonagreement, 2.21; *P *= 0.02) [27]. Another study showed that accuracy of measurements with glucose meters by using arterial blood samples were significantly varied among devices (incidence of nonagreement (StatStrip = reference): Accu-Chek Inform: odds ratio, 5.2; *P *< 0.001, Precision PCx: odds ratio, 15.2; *P *< 0.001; SureStepFlexx, odds ratio, 4.3; *P *< 0.001) [28].

**Table 6:** Three studies in which agreement of criteria other than "within 20%" was assessed

	First author (year)	Devices	Methods for assessment	Nonagreement proportion < 5%	Nonagreement proportion	Ref
Gluco-C	Cook A (2009)	SuperStepFexx	Within 20 mg/dl	No	10/64 (15.6%)	[26]
	
	Karon BS (2007)	Accu-Chek inform	Within 10%	No	25/96 (26.0%)	[27]

Gluco-A	Karon BS (2007)	Accu-Chek inform	Within 10%	No	42/96 (43.8%)	[27]
	
	Karon (2008)	Accu-Chek Infor Precision PCx SureStepFlexx StatStrip	Within 10%	No	58/185 (31.4%) 106/185 (57.3%) 51/185 (27.6%) 15/185 (8.1%)	[28]

## Discussion

Although several reviews focused on the accuracy of point of blood-glucose monitoring in critically ill patients [10,37,38], our review is the first systematic review for this issue. Our review shows comparisons among devices and between hypo- and non-hypoglycemic ranges, as well as problems in studies including variation of references and insufficient data for a hypoglycemic range.

Although available data are often heterogeneous and insufficient for meta-analysis, we found that the accuracy of blood-glucose monitoring might vary, especially according to the device, site of blood sampling, and glucose range. With our systematic analysis of the 11 retrieved articles, we considered that, despite the limitation of data, some statements can be made to help establish current knowledge of the accuracy of point of blood-glucose monitoring in critically ill adult patients.

### Statement 1: Type of central laboratory machine (reference) is highly variable

The type of central laboratory machine varied among the studies. Although all central machines used in the 11 studies have traceability of blood-glucose monitoring, it is unclear whether these machines are equally accurate. Thus, it is difficult to interpret whether the type of laboratory machine influenced the accuracy of point of blood-glucose monitoring. If the central laboratory machine does not have metrologic traceability for blood-glucose monitoring, it should be the case for quality-insurance programs requirements.

### Statement 2: In few studies was the accuracy of ABGs compared with that of a glucose meter simultaneously

In the variation of reference as in statement 1, the study to compare the accuracy among Glu-ABGs, Gluco-C, and Gluco-A is essentially relevant. However, in only three studies were the accuracies of these three compared [18-20].

### Statement 3: Accuracy of ABG analyzers might vary among devices

The proportion of nonagreement in Glu-ABGs varied widely (0 to 42.8%). Although five of the six studies showed good quality of Glu-ABGs, and the range of limits of agreements for Glu-ABGs (minimum of 19 mg/dl, maximum of 39 mg/dl) was smaller than those for Gluco-C and Gluco-A, one study showed overestimation by Glu-ABGs in 42.8% of the samples. Although it is unclear whether the type of central laboratory machine, conditions of the measurement, or other unknown mechanisms affected the results of that study, the results suggested that accuracy of Glu-ABGs might vary among devices. Thus, it is recommended that each institution confirm the accuracy of their ABGs for blood-glucose monitoring.

### Statement 4: ABGs and a glucose meter using arterial blood were significantly more accurate than a glucose meter using capillary blood

Glu-ABGs and Gluco-A were significantly more accurate than Gluco-C. Even when we included studies using criteria other than within 20%, the finding did not change (odds ratio for nonagreement, 0.43; *P *= 0.01). Thus, for blood-glucose measurements in critically ill adult patients, arterial blood samples should be used rather than capillary blood samples.

### Statement 5: Blood-glucose monitoring with ABG analyzers tends to be more accurate than that with glucose meters using arterial blood

Our meta-analysis showed that Glu-ABGs tend to be more accurate than Gluco-A (*P *= 0.20). Additionally, the range of limits of agreements in Glu-ABG was smaller than that in Gluco-A. These results suggest that Glu-ABGs might be more appropriate than Gluco-A.

However, it should be noted that the accuracy of Gluco-A varied among studies, as stated earlier, and in only three studies were they compared, and the results were conflicting (odds ratios for error, 0.03 to 3.00). Thus, further studies are needed to determine whether Glu-ABGs, Gluco-A, or both can be recommended for blood-glucose monitoring in a critically ill setting.

### Statement 6: Information on the accuracy of blood-glucose measurement in the hypoglycemia range is not sufficient

Although more than 6,000 samples were assessed for the accuracy of blood-glucose measurements (ABG, 1,360; Glu-C, 2,858; Glu-A, 3,086), about 70 samples were in the hypoglycemic range in each method (ABG, 58; Glu-C, 77; Glu-A, 81). This number of samples is not sufficient to compare between devices and determine the risk factors of error. Therefore, further studies are needed for blood-glucose measurements in the hypoglycemic range.

### Statement 7: Blood-glucose monitoring in the hypoglycemic range is less accurate than that in the nonhypoglycemic range

Because many studies have shown that even mild hypoglycemia is significantly associated with increase in mortality [39,40], accuracy of blood-glucose monitoring in the hypoglycemic range is important. Although little information is available for the hypoglycemic range, as stated earlier, our results showed that the incidences of errors in the hypoglycemic range were higher than those in the nonhypoglycemic ranges.

Regardless of the method used for blood-glucose monitoring, we should be aware that a greater possibility of errors exists in the hypoglycemic range than in the nonhypoglycemic range. We should confirm blood glucose concentrations by using Glu-lab when we obtain blood-glucose values within or near the hypoglycemic range.

### Statement 8: Unstable hemodynamics and insulin infusion might increase the risk of errors in blood-glucose monitoring by using a glucose meter

Unstable hemodynamics (low perfusion index, use of a vasopressor, presence of edema, and low mean arterial pressure) and insulin infusion were associated with increased risk of inaccuracy. These factors might decrease peripheral blood-glucose concentrations through microcirculatory disturbance and increased tissue glucose consumption [41,42]. Therefore, physicians should avoid using either Gluco-A and Gluco-C in patients with unstable hemodynamics and/or receiving insulin infusion.

### Limitations

Our systematic review has some limitations. Our literature search was performed by using only MEDLINE and PubMed and was performed by only one author. The use of other important databases, such as the Cochrane systematic reviews database, and selection by multiple authors might have made the literature review more comprehensive. We also excluded non-English-language reports, abstracts, and unpublished studies. Thus, some findings may have been missed. However, the selection was done with preset inclusion criteria and a careful search of bibliographies so as to minimize selection bias.

## Conclusions

Our literature review showed that ABGs were significantly more accurate than glucose meters using capillary blood and tended to be more accurate than glucose meters using arterial blood. However, these results should be interpreted with caution because of the large variation of accuracy among devices. Because blood-glucose monitoring was less accurate within or near the hypoglycemic range, especially in patients with unstable hemodynamics or receiving insulin infusion, we should aware that current blood-glucose monitoring technology has not reached a high enough degree of accuracy and reliability to lead to appropriate glucose control in critically ill patients.

## Key messages

• Accuracy of blood-glucose measurements using arterial blood gas analyzers might vary among devices.

• Blood-glucose monitoring with ABG analyzers tends to be more accurate than that by glucose meters with arterial blood.

• Arterial blood samples should be used rather than capillary blood sample for blood-glucose measurements in adult critically ill patients.

• In the hypoglycemic range, blood-glucose monitoring is more inaccurate than that in the nonhypoglycemic range.

• Unstable hemodynamics and insulin infusion might increase the risk of error in blood-glucose monitoring with a glucose meter.

## Abbreviations

ABGs: arterial blood gas analyzers; Glu-ABGs: blood-glucose measurements by ABGs; Gluco-A: blood-glucose measurements with glucose meters by using arterial blood samples; Gluco-C: blood-glucose measurements with glucose meters by using capillary blood samples; Glu-lab: central laboratory blood glucose measurements by using plasma; ISO: International Organization for Standardization.

## Competing interests

The authors declare that they have no competing interests.

## Authors' contributions

ME and MK conceived the study. ME, JK, and MK participated in the design of the study. SI performed systematic literature search. SI and ME retrieved relevant information from selected articles. SI and ME performed the statistical analyses. SI, ME, JK, and KM participated in data interpretation and drafted the manuscript. All authors read and approved the final manuscript.
